# The badness of death and priorities in health

**DOI:** 10.1186/s12910-016-0104-6

**Published:** 2016-04-14

**Authors:** Carl Tollef Solberg, Espen Gamlund

**Affiliations:** Department of Global Public Health and Primary Care, University of Bergen, PB. 7804, 5018 Bergen, Norway; Department of Philosophy, University of Bergen, PB. 7805, 5020 Bergen, Norway

**Keywords:** Age weighting, Badness of death, Welfare loss, Discounting, Deprivation account, Epicureanism, Global burden of disease, Health priorities, Morbidity, Time-relative interest account

## Abstract

**Background:**

The state of the world is one with scarce medical resources where longevity is not equally distributed. Given such facts, setting priorities in health entails making difficult yet unavoidable decisions about which lives to save. The business of saving lives works on the assumption that longevity is valuable and that an early death is worse than a late death. There is a vast literature on health priorities and badness of death, separately. Surprisingly, there has been little cross-fertilisation between the academic fields of priority setting and badness of death. Our aim is to connect philosophical discussions on the badness of death to contemporary debates in health priorities.

**Discussion:**

Two questions regarding death are especially relevant to health priorities. The first question is why death is bad. Death is clearly bad for others, such as family, friends and society. Many philosophers also argue that death can be bad for those who die. This distinction is important for health priorities, because it concerns our fundamental reasons for saving lives. The second question is, ‘When is the worst time to die?’ A premature death is commonly considered worse than a late death. Thus, the number of good life years lost seems to matter to the badness of death. Concerning young individuals, some think the death of infants is worse than the death of adolescents, while others have contrary intuitions. Our claim is that to prioritise between age groups, we must consider the question of when it is worst to die.

**Conclusions:**

Deprivationism provides a more plausible approach to health priorities than Epicureanism. If Deprivationism is accepted, we will have a firmer basis for claiming that individuals, in addition to having a health loss caused by morbidity, will have a loss of good life years due to mortality. Additionally, Deprivationism highlights the importance of age and values for health priorities. Regarding age, both variants of Deprivationism imply that stillbirths are included in the Global Burden of Disease. Finally, we suggest that the Time-Relative Interest Account may serve as an alternative to the discounting and age weighting previously applied in the Global Burden of Disease.

## Background

It is evident that longevity is not equally distributed across the world [1]. The state of the world is also one of scarce medical resources. Given such facts, setting priorities in health entails making difficult yet unavoidable decisions about which lives to save. Moreover, the business of saving (i.e., extending) lives works on the assumption that longevity is valuable and that an early death is generally worse than a late death. There is a vast literature on health priorities and badness of death, separately. However, there has been little cross-fertilisation between these academic fields. Our primary aim is to connect philosophical discussions on the badness of death to contemporary debates in health priorities. More precisely, we will show the relevance of the following two questions for priorities in health: Why is death bad? When is the worst time to die?

This paper proceeds as follows. First we outline a case of illustration that sets the stage for the subsequent discussion. Next we discuss the question of why death is bad. Here we begin by introducing some relevant distinctions, and then we present two competing theories on the badness of death, Epicureanism and Deprivationism. We defend Deprivationism and suggest some implications of this view for health priorities. In the final part we discuss the question of when is the worst time to die. Here we present two variants of Deprivationism, one continuous and one discontinuous. We show the relevance of these two views for health priorities, and suggest that a continuous view is preferable to a discontinuous one.^1^

## Discussion

### A case of illustration

Before we begin, we make three central assumptions. First of all, that death is followed by permanent non-existence. Secondly, we presume that there are goods and evils in life (although we do not presuppose a particular theory of welfare). Thirdly, that every individual saved will live until the age of 86, and those not saved will die within a short time-span. Moreover, we discuss health priorities from a population perspective, and we will use the following hypothetical priority case to set the stage for our discussion. In Table 1, we present six age groups where each group comprises a thousand individuals.

**Table 1:** Each group comprises 1000 individuals. The groups saved will live an average of 86 years with a similar average quality of life. Those not saved will die within a short time-span. This is in line with the Global Burden of Disease

Group	Age	Moral principles				
A	20-week fetuses	YF	FI			
B	Infants	YF	FI	GB		
C	5 years	YF	FI	GB		
D	15 years		FI	GB	MF	SV
E	30 years		FI	GB	MF	SV
F	70 years					

We note that this case of illustration contains several *ceteris paribus* claims. This is necessary in order to focus attention on how different moral principles work and guide our decisions about which lives to save

*Abbreviations: YF* youngest first, *MF* modified youngest first, *FI* fair innings, *SV* societal value, *GB* greater benefit

Suppose first that we have to choose between E and F. Here, most people would choose E. One reason is that the 30-year olds would gain a greater benefit from being saved. This is in accordance with the greater benefit principle, which states that resources should be accorded to the intervention with the greater health benefit [2]. Moreover, the 30-year olds will be more productive, able to reproduce, and will have people who are dependent on them such as children and parents. Call this the societal value principle. Another reason for prioritising E is that the 30-year olds have had fewer years of life than the 70-year olds. This is in line with a standard version of the fair innings principle, according to which resources should be directed to those who have not yet had their fair share of life [3, 4]. Group E will also draw support from a modified youngest first principle, according to which extra priority should be given to individuals between 15 and 40 years of age [5]. The principles mentioned explain our intuitions about the trade-off between E and F.

But suppose we have to choose between saving B and D. How do the principles apply in this case? The youngest first principle and the greater benefit principle would support saving B over D. According to one version of the fair innings principle, it is of moral importance that the infants have lived fewer years than the 15-year olds, and so have had less of their fair share. However, both a modified youngest first principle and a societal value principle would support prioritising D over B. The question is how these different principles should be weighed against each other? For example, how important is societal value in the trade-off between B and D? On the assumption that all these principles are relevant, we get a weighing problem. We will not attempt to solve this problem.

### Why is death bad?

All health care systems share two basic goals: saving lives and improving the quality of life. The first goal gives rise to two essential questions: (i) Why should we save lives? (ii) Which lives should we save first? In the health priorities literature, the second question has received the most attention. We believe (i) and (ii) are closely connected, and that an answer to (ii) presupposes an answer to (i). In order to make claims about which lives to save first, we need an account of why we should save lives in the first place. One justification for saving lives is simply that death is bad. Saving lives entails postponing death, which is justified on the assumption that an early death is worse than a late death. One could, however, argue that we should justify saving lives with reference to considerations of fairness. Although we do not deny this, our aim is a different one, namely that of investigating the reasons we have for saving lives that stem from considerations of the badness of death.

We will briefly clarify the concept of death before we proceed. “Death” can refer to at least four dimensions: “the prospect”, “the process”, “the incident” and “the loss”. The *prospect* refers to our knowledge of being mortal, which as far as we know is unique to human beings. The *process* of dying is an event that may be filled with pain, as in some instances of cancer, or it may happen abruptly, as in a traffic accident. The *incident* of death is when someone goes from existence to non-existence. Finally, there is a permanent *loss* when death occurs because there is no future for that individual.

Although many tend to focus on the process of dying, our focus will be on the loss. Arguably, if dying had not been followed by permanent non-existence, then perhaps dying would not be so bad after all. Interestingly, the *loss* dimension of death seems to play an important role in current health priorities debates. One example is the estimation of health loss due to both morbidity and mortality in traditional cost-effectiveness analyses; another is the Global Burden of Disease project [6]. A third example is two recent articles by Ezekiel Emanuel and Govind Persad, which we discuss shortly.

If the loss dimension is accepted, the question is for whom death represents a loss. There are two rival theories to this question: *Epicureanism* and *Deprivationism*. Epicureanism refers to a contemporary view on the badness of death inspired by the ancient philosopher Epicurus, which states that death is not bad for those who die [7–9]. Both theories are compatible with the idea that death can represent a loss for others (such as family, friends, and society), but only Deprivationism accepts that death represents a loss for those who die [10–19]. First, we discuss Epicureanism and priority setting implications, followed by a discussion of Deprivationism.

#### Epicureanism

The two arguments normally offered in favour of Epicureanism are the experience argument and the time argument. The experience argument is best illustrated by the expression, “What you don’t know won’t hurt you”. One interpretation of this is that in order for something to be good or bad for us, we must experience its goodness or badness. But of course when we are dead, we cannot experience. Therefore, death cannot be good or bad for us.

There are, in fact, cases within life where something is good or bad for us, even if we cannot experience it. Roughly speaking, we can imagine two types of such cases, moral and medical. Examples of moral cases are infidelity, lying, tale bearing, and stealing. It can be argued that such actions are wrong independently of whether those affected by cheating and lying will experience this [17]. Examples of medical cases are prevention, loss of senses, coma, asymptomatic diseases and risk factors. For instance, it seems clear that having cancer is bad for a patient even before the cancer is detected.^2^ If we accept that there are goods and evils within our lives that we cannot experience, we are left with no reason to deny that death can be a similar evil.

The time argument states that for something to be good or bad for us, there must be a time at which things are good or bad for us. But death is not good or bad for us while being alive. Nor is death good or bad for us while being dead. Since there is no time at which death is good or bad for the person who dies, death cannot be good or bad for this person [19].

There are at least four views one can adopt in responding to this argument. One view is that death is bad before it occurs, another is that death is bad when it occurs, a third is that death is bad after it occurs, and a fourth is that death is bad at a time which cannot be easily identified. One can successfully object to the time argument on the basis of one of these four views. We believe the fourth view is the best strategy for responding to the time argument. Here are some cases of analogy in support of the fourth view. For example, never having an education, freedom, or children can be bad even if its badness cannot be ascribed to a specific time. Moreover, at times, people may be grateful for not being a victim of accidents or suffering from severe sickness, even if “the evils that they never suffered” cannot be so easily located in time. Finally, consider a case of prevention. Somewhat paradoxically, prevention works when nothing happens. When, for instance, a vaccine proves successful, the individual does not suffer the disease in question. The good that follows from not suffering the disease does not occur at a particular time.^3^ If these examples are accepted, it follows that there are goods and evils within life that cannot be so easily located in time. In our view, death is an analogous evil in this sense.

If one accepts either the experience- or the time argument, it follows that death cannot be bad for those who die. What does this imply with regard to health priorities? If death is no loss for those who die, it matters less whether we suffer a premature or a late death. Consequently, age will play a less significant role (if any role at all) to health priorities. With Epicureanism we are, however, left with the option that death is bad for third parties such as family, friends, and society. This implies a higher emphasis on saving lives for the sake of others. Moreover, this suggests that what matters from a moral point of view are things like the emotional attachments and investments of family, friends, and society. In addition, the death of individuals can be bad by virtue of being a loss of caring relationships, productivity, or simply in terms of the world being deprived of a person.

While these considerations are no doubt important, none of them concern the individuals who stand at risk of dying prematurely. But this is clearly problematic. Epicureanism would still fail to account sufficiently for those who are already orphans, those without friends and people who are not productive in society. More precisely, it fails to account for our obligations to save individuals’ lives for their own sake. In this sense, Epicureanism would be radically different from current priority practice, where we primarily save lives for the sake of the individual whose life it is. In view of these remarks, Epicureanism is vulnerable to important objections and fails to capture everything we care about when saving lives.

#### Deprivationism

In this part, we offer a positive account of Deprivationism. This account is inspired by Thomas Nagel, who says that, ‘If death is an evil at all, it cannot be because of its positive features, but only because of what it deprives us of’ [17]. Some things in life can be good or bad in themselves, such as pleasure and pain. Death, on the other hand, is a different kind of evil. Suppose you suffered from paralysis in both your legs as a result of an accident. This accident deprives you of the chance to do a lot of things, like walking or playing tennis. In a similar way, death deprives us of the opportunity to continue with our lives. And assuming that continued life contains value, death is bad for us. Deprivationism explains how we can make judgments concerning the badness of death by comparing at least two different outcomes: (a) how well off individuals would have been if they continued to live and (b) how good it is for individuals not to continue with their lives. As long as (a) is better than (b), death is an evil. In some cases (b) might be better than (a), in which case death is not necessarily an evil [15].

Deprivationism is the standard view on the badness of death. We suggest that Deprivationism is relevant to health priorities in at least four areas. First, Deprivationism brings attention to the kinds of values that are lost when death occurs. Secondly, it emphasises that age matters. Thirdly, Deprivationism will favour a person-affecting theory.^4^ Fourthly, it may say something new about who the worst off are.^5^ Jointly these four areas can provide reasons for saving lives. In what follows, we discuss the first two areas of relevance in more detail.

With regard to values, it should be clear that they are lost when death occurs. But exactly what kinds of values are lost? Philosophers typically discuss the values that occur within our lives, with less attention being given to the values that death deprives us of. Here, we highlight some categories of values that are important when thinking about death. One dimension is value for others, such as ‘societal value’. This type of value has two dimensions, a wide and a narrow one. Call wide societal value the value individuals have or can have for society, for example, productivity and societal investments. Call narrow societal value the value individuals have or can have for friends and family. Importantly, Deprivationism highlights a second value dimension, namely the value of the future of an individual if he or she is saved. Call this ‘personal value’, which in our context refers to the values lost when individuals die.^6^

When a death occurs we tend to focus on the loss of the good or bad conditions present in us, such as knowledge, language, and memory. This is undoubtedly important, but death also causes a loss of future possibilities. This entails a focus on good or bad potential future states of individuals. It includes everything the future might hold for them, such as having intimate relationships, having aesthetical experiences, and simply enjoying the pleasures of life. In this sense, death is bad to the extent that it deprives individuals of personal value. If one accepts that personal value is important, and that personal value is lost when death occurs, then it follows that we have reasons to prevent death or save lives for the sake of those whose life it is.

Regarding age, there is an on-going debate about whether it should matter to health priorities at all, indirectly or as an independent criterion [20]. We suggest that age indicates something about three important dimensions: life stage, years lived, and future life years. Deprivationism focuses on life stage and future welfare. Life stage is important because it says something about the extent to which individuals have ownership to their future. Future life years indicate the potential future individuals can have. Moreover, if we accept either life stage or years lived, age will matter. This means that if we accept Deprivationism, it follows that age is morally relevant to health priorities. In any case, it seems that age will matter somehow to priorities in health.

### When is the worst time to die?

Though the idea that age matters to health priorities has gained a certain acceptance, there is bound to be disagreement about which age groups to prioritise. This issue is the subject of contemporary debate. Since the launch of the Millennium Development Goals, mother and child campaigns have been high on the agenda. The goal of reducing under-five mortality has received special attention. In 2015, we got new Sustainable Development Goals that replaced the Millennium Development Goals [21]. Against this background, one important question is whether to give special attention to infants and young children, on the one hand, or to adolescents and young adults, on the other. Our claim is that in order to prioritise between age groups, it is relevant to consider the question of when it is worst to die.

As a starting point, we propose to consider some claims about death drawn from the health priorities literature:

“It is terrible when an infant dies, but worse, most people think, when a three-year-old child dies and worse still when an adolescent does” [22].

“While every premature death is distressing, death in childhood is particularly tragic, as children lose more future years and stages of life than adults” [23].

“The death of a 20-year-old young woman is intuitively worse than that of a 2-month-old girl, even though the baby has had less life” [5].

The second paragraph states the intuition that death is worse the earlier it occurs. In this sense, Colleen C. Denny & Ezekiel J. Emanuel support a youngest first principle [23]. In their article, they motivated a greater focus in the USA on prioritising mothers and young children. The first paragraph presents the intuition that the death of adolescents is worst, whereas in the third paragraph it is assumed that the death of young adults is worse than the death of young children. The intuitions stated in the first and third paragraphs support a modified youngest first principle while the greater benefit principle is here given less weight. The paragraphs demonstrate three things. First of all, intuitions about death play a role in justifying certain priority principles. Secondly, such intuitions diverge on the issue of when it is worst to die. Thirdly, the authors are silent on the distinction between death as bad for those who die and death as bad for others.

When intuitions conflict, as in the question of when it is worst to die, reasoning becomes especially important. To this end, Deprivationism can provide theoretical support. To begin with, an early death is worse than a late death. Age and the number of good life years lost matter to the badness of death. Different theories exist about the badness of death. Some theories consider the death of infants worst, while others consider the deaths of young individuals to be worst. We will focus on deprivationist theories, which comes in *continuous* and *discontinuous* forms. The *Deprivation Account* is an example of a discontinuous theory, whereas the *Time-Relative Interest Account* is an instance of a continuous theory.

#### The deprivation account

The standard view on Deprivationism is the Deprivation Account. According to this account, death is generally worse the more good life years it deprives us of. This implies that death is worse the earlier it occurs. Given this claim, some have questioned the plausibility of the Deprivation Account. Ronald Dworkin, for instance, believes this account amounts to what he calls ‘the simple loss view’. According to Dworkin’s interpretation of the Deprivation Account, the badness of death is only a function of the amount of life lost when someone dies. Applied to our case of illustration, this would favour saving group A (20-week fetuses). Dworkin claims that this is counterintuitive. He argues that if the loss of life years were the only thing that mattered, then an early stage abortion would be a worse than a late stage abortion. He points out that almost everyone holds the contrary assumption; late stage abortion is worse than early stage abortion. Thus, there is a conflict between the simple loss view and people’s intuitions [22].

**Fig. 1:**
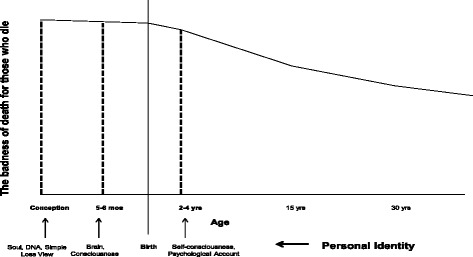
The Deprivation Account and personal identity. The figure illustrates how the Deprivation Account relies on personal identity from a population perspective. This is not a priority curve, but rather a badness of death curve. There are actually three graphs in one, each represented by a stipulated line. The y-axis represents the badness of death for those who die. The x-axis represents age with an emphasis on the fetal life. Each vertical line represents a view on personal identity. Moreover, these vertical lines illustrate the discontinuity of the Deprivation Account

Why is the simple loss view incorrect? On Dworkin’s account, it is incorrect because it only focuses on future possibilities. It is, however, necessary to emphasise that the simple loss view is only one variant of the Deprivation Account, something Dworkin overlooks. More precisely, he fails to capture the discontinuous nature of the Deprivation Account. It is worth noting that it is not only the loss that matters, but also to whom the loss belongs. If we consider “those who never existed” due to infertility and contraceptives, such “losses” are not equivalent to death. In order for a loss to be personal, the future must belong to the one who dies. Those who subscribe to the Deprivation Account must explain the difference between the loss associated with “those who never existed”, and the loss associated with death. They will need to rely on a notion of personal identity to explain this difference. Personal identity refers to those properties necessary to make an individual at t_1_ numerically identical with himself at t_2_ [12]. In this sense personal identity gives someone “ownership” to his loss of life. In Fig. 1, we have tried to illustrate the importance of personal identity for the Deprivation Account.

Since there are different views on personal identity, there are different variants of the Deprivation Account. The crucial question for this account is therefore when we acquire personal identity (i.e., when we begin to exist). Before personal identity is acquired, death represents no loss. As soon as personal identity is acquired, death is the greatest loss. The figure shows some possible views on personal identity: soul, brain, consciousness, and self-consciousness. For example, if a soul (or DNA) is what grants personal identity, then death is the greatest evil right after conception. This, however, conflicts with widely held views on termination of pregnancy, spontaneous abortions, and the use of in-vitro fertilization clinics.

Our aim here is not to defend any particular conception of personal identity; nevertheless, it is clear that some conceptions are preferable to others. For example, in order for Dworkin’s interpretation of the Deprivation Account to be correct (i.e., the simple loss view), he would have to argue that personal identity is acquired at conception. As far as we know, Dworkin does not provide such an argument in his work. Moreover, there are good reasons for doubting that personal identity is acquired at conception. First of all, it is reasonable to believe that humans cannot be individuated before the point in development when twinning could occur. Available evidence suggests that twinning can occur within two weeks after conception [24]. Furthermore, it is unresonable that DNA grants personal identity since separate individuals can have the same DNA, such as monozygotic twins and dicephalic twins. Secondly, there exist convincing arguments against a soul view [12].

Finally, very few defenders of the Deprivation Account claim that personal identity is acquired at conception. Given these remarks, some of the standard criticism of the Deprivation Account may be off target [12]. This includes the criticism by Dworkin and that related to abortion [22]. Moreover, very few believe that self-consciousness is what gives us personal identity. On the most promising accounts, personal identity is acquired between five months of fetal life and birth [12, 17]. Thus, death is the greatest evil in this time period. The individuals in question will lose the most good life years, and they will (in virtue of acquired personal identity) possess an ownership to that loss.

How exactly is the Deprivation Account relevant to health policy? One case in point is the Global Burden of Disease study. In the most recent version of this study, birth is treated as morally significant. Death right after birth generates 86 Disability Adjusted Life Years (DALYs), whereas death right before birth generates 0 DALYs. Thus, the study implies that preventing the death of infants is the most important health intervention, while preventing stillbirths is of little (if any) importance. Inevitably, a study like the Global Burden of Disease comes with certain normative presuppositions. This is not necessarily problematic in itself. However, the treatment of birth as morally significant, is problematic and in need of a defence. Although such a claim might find philosophical support, to our knowledge no such view is defended in the badness of death literature [25]. Given the framework that we discuss here, the Global Burden of Disease study might gain theoretical support from the Deprivation Account. But this would imply the inclusion of stillbirths.

One may ask whether personal identity is relevant to the question of which lives to save. An important criticism of the Deprivation Account emphasises the fact that it relies on personal identity. For one thing, the concept of personal identity depends on metaphysical presuppositions that many regards with suspicion. For another, even if we accept the concept of personal identity, there is bound to be disagreement on how the concept should be understood. This suggests that from a pragmatic point of view, one can question whether a reliance on personal identity is helpful in the context of setting priorities in health. In summary, such criticism of the Deprivation Account has motivated the development of an alternative continuous view on the badness of death.

#### The time-relative interest account

**Fig. 2:**
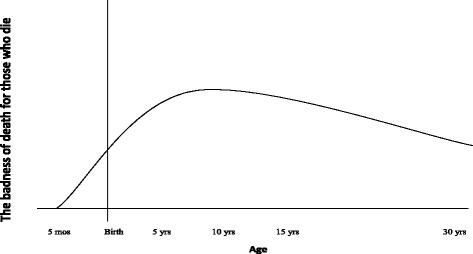
The Time-Relative Interest Account. This is our interpretation of the Time-Relative Interest Account applied on a population perspective. Again this is not a priority curve, but a badness of death curve. The y-axis represents the badness of death for those who die. The x-axis represents age, once again with an emphasis on fetal life. The curve peaks somewhere around ten years of age. In our figure, we suggest a grading from 0 to 1, where 0 is no ownership and 1 is full ownership

In our view, the most promising continuous view at the moment is Jeff McMahan’s *Time-Relative Interest Account* [12, 26]. Following this account, death is an evil by virtue of two factors: the number of good life years lost (similar to the Deprivation Account), and how psychologically connected one is to the future that is lost. On this account, personal identity is not what matters to the badness of death; rather it is individuals’ psychological development. What binds us to the future in a morally relevant way are direct psychological connections—such as memory, language, beliefs, intentions, expectations, values and knowledge—and the continuity of those connections [12]. Such direct psychological connections obviously come in degrees. Since psychological connectedness is a matter of degree and is what grants ownership to the future, such ownership will have to come in degrees. In Fig. 2, we present our interpretation of the Time-Relative Interest Account. Notably, this curve is continuous because, according to this account, ownership to the future is graded in accordance with age, understood as the life stage of individuals.

According to the Time-Relative Interest Account, psychological connectedness starts with the development of a brain. By approximately six months of fetal life, such connections are very weak.^7^ The value of psychological connectedness goes from zero and up to a threshold. By the time individuals reach approximately ten years of age, they have reached a threshold in the sense that their psychological connections have become sufficiently strong to ground a complete ownership to the future.

What does this imply for our case of illustration? We believe that the degree of ownership to the future might serve as a weighting function for the welfare loss caused by death. If this is accepted, the Time-Relative Interest Account implies that those in Group D have complete ownership of their future. Since individuals older than 10 years of age have complete ownership, the Deprivation Account will account for the badness of death for individuals older than 10 years of age. If the badness of death for those who die were the only thing that mattered to health priorities, it would mean that Group D should be favoured over the other groups. The Time-Relative Interest Account does not necessarily guide us in choosing between Groups B and E, but both groups seem to be favoured over Group F. The Deprivation Account, on the other hand, would clearly favour Group B. Group A is given least priority by both the Deprivation Account and the Time-Relative Interest Account because those in this group lack a morally relevant connection to their future. As pointed out by Jeff McMahan, it is as “if the future it loses might just as well have belonged to someone else” [12].

Returning to the Global Burden of Disease, there is considerable debate as to whether future health benefits should be discounted, and if so, why they should be discounted. Three reasons are often mentioned in favour of discounting. First of all, the future is surrounded by uncertainty; secondly, people have weaker preferences for future goods; and finally, future health interventions will improve [27]. In some previous studies of the Global Burden of Disease, DALYs have been time-discounted and age-weighted. Many are sceptical, however, that a theoretical foundation for discounting future health benefits can be offered. Even so, it should be noted that the badness of death curve for the Time-Relative Interest Account would be similar to a time-discounted and age-weighted DALY curve, albeit for different reasons that was used in previous studies of the Global Burden of Disease [28].

The Time-Relative Interest Account can offer a philosophical foundation for a similar Global Burden of Disease curve without relying on discounting or age weighting in the traditional sense. As mentioned, the Time-Relative Interest Account weights future benefits in accordance with the psychological connectedness individuals are expected to have according to life stage. Of course, when discussing priorities in health we are in fact discussing reductions in both morbidity and mortality. By taking into account reductions in morbidity, matters become complicated when considering the implications of the Time-Relative Interest Account. Our suggested weighting function for future health benefits according to age is with regard to mortality only. The curve in Fig. 2 peaks somewhere around ten years of age. In this figure, we suggest a grading from 0 to 1, where 0 is no ownership and 1 is full ownership. As an alternative to time discounting, the suggested grading might serve as a weighting function for DALYs.

What does this imply with regard to saving lives? Suppose we must prioritise between Group B and Group D in our illustrated case. Following the Deprivation Account, we should save Group B since 86 DALYs are generated when infants die, whereas only 71 DALYs are generated when 15-year-olds die. Following the Time-Relative Interest Account, on the other hand, we should save Group D (15-year olds). This is because the individuals in Group B do not have complete ownership to their future, whereas the individuals in Group D have acquired complete ownership. This might be formalised as follows: infants 86 DALYs × 0.5 = 43 weighted DALYs, whereas 15-year olds 71 DALYs × 1 = 71 weighted DALYs. More thinking is needed on how the weighting rates should be operationalized. Still, we believe we have shown that the Time-Relative Interest Account implies some form of weighting.

### Conclusions

In this paper, we have discussed different theories on the badness of death and showed some of their implications for health priorities. We have introduced an important distinction between the badness of death for others and the badness of death for those who die. Our conclusion is that Deprivationism provides a more plausible approach to health prioritisation than Epicureanism. If Deprivationism is accepted, we will have a firmer basis for claiming that individuals, in addition to having a health loss caused by morbidity, will have a loss of good life years due to mortality. Deprivationism highlights the importance of age and values for health priorities. With regard to age, both variants of Deprivationism imply that stillbirths should be included in the Global Burden of Disease. Finally, we suggest that the Time-Relative Interest Account may serve as an alternative to the discounting and age weighting previously applied in the Global Burden of Disease. We consider this work to be a first step and have suggested some starting points for further debate. It is our hope that this discussion can stimulate more thinking on how Deprivationism might be strengthened and implemented in health priorities.

## Footnotes

1 This paper builds on some ideas that we have developed previously in a Norwegian paper. See [29].

2 The following case can serve as an objection to our argument. Suppose that A and B both have asymptomatic cancer. A’s doctor tells A, whereas B’s doctor does not tell B. One may ask whose life is better between the time of diagnosis and the time at which B begins to experience symptoms? Some would claim that B’s life has been better, because knowing that he had asymptomatic cancer has psychologically harmed A. Thanks to Alan Wertheimer for pointing this out.

3 As pointed out to us by Guy Schofield, it might be that vaccination is a good at the time one is re-exposed to the agent from which one was vaccinated. In response to this, we think it is important to distinguish between (a) the causal effect of the vaccine, and (b) the benefit from the vaccination. Although causal effects occur at specific times, it does not follow that the benefit from the vaccination – in the sense of the disease one never got – can be ascribed to particular times in the same way.

4 Deprivationism seems to emphasise what is called a ‘person-affecting claim’, which means that nothing can be bad if it is bad for no individual. We think Deprivationism can be relevant to the trade-off between extending existing lives and creating new lives, i.e., extending existing lives is more important than creating new ones.

5 The ‘worst off’ may refer to the sickest, either currently or in the past, or it may refer to those who have lived the shortest lives. With reference to the badness of death, the worst off may be those who suffer the greatest loss. There is also a question about how to understand the concept of being worst off. For example, there is an on-going discussion about whether it is possible to be worst off in an absolute sense. Some argue that we can only be worse off in a relative sense. Our hypothesis is that Deprivationism, by emphasising intra-individual counterfactual reasoning, may provide an original argument for an absolute conception of the worst off.

6 Our notion of personal value does not presuppose any specific theory of welfare. It is compatible with hedonism, preference satisfaction theory, and objective list theories. It is important to note that we are interested in welfare in a personal rather than an impersonal sense.

7 It is, of course, an empirical question when the neurogenesis begins. Moreover, there is currently disagreement on exactly when awareness is realised in the fetal life.
